# Supplementary material for: Alternative climatic steady states near the Permian–Triassic Boundary
Source: Sci Rep. 2024 Oct 30;14:26136. doi: 10.1038/s41598-024-76432-8 (PMC11525819; doi:10.1038/s41598-024-76432-8)
Supplement: Supplementary file 1 — Supplementary Information. [file 41598_2024_76432_MOESM1_ESM.pdf]

Supplementary Information:

Alternative climatic steady states near the Permian-Triassic Boundary

C. Ragon · C. Vérard · J. Kasparian · M. Brunetti*

[1] Group of Applied Physics and Institute for Environmental Sciences, University of Geneva, 66 Bd Carl-Vogt, CH-1211 Geneva 4, Switzerland

[2] Section of Earth and Environmental Sciences, University of Geneva, 13 Rue des Maraîchers, CH-1205 Geneva, Switzerland

* Corresponding author: Maura Brunetti, maura.brunetti@unige.ch

1 Supplementary material on the three climatic attractors

We described in the main text the asymmetry between the northern and southern hemispheres in the mean annual atmospheric overturning circulation (Fig. 3a-c). Here, we describe the solstitial patterns, where the cell of the winter hemisphere becomes the only vigorous one (Lindzen and Hou 1988; Dima and Wallace 2003).

The trend observed in the annual mean, as depicted in Fig. 3 in the main text, is confirmed by the seasonal circulation, shown in Fig. S1: the austral winter cell (Fig. S1a-c) is slightly wider and weaker in the hot state; the boreal winter cell, with similar width in all attractors, is stronger and more shifted towards the Equator in the cold state, as shown in Fig. S1d-f.

The sea surface salinity is shown in Fig. S2 for the three attractors. In the hot state, where sea ice is absent, the salinity distribution is symmetrical with respect to the Equator, as is the sea surface temperature (Fig. 1d in

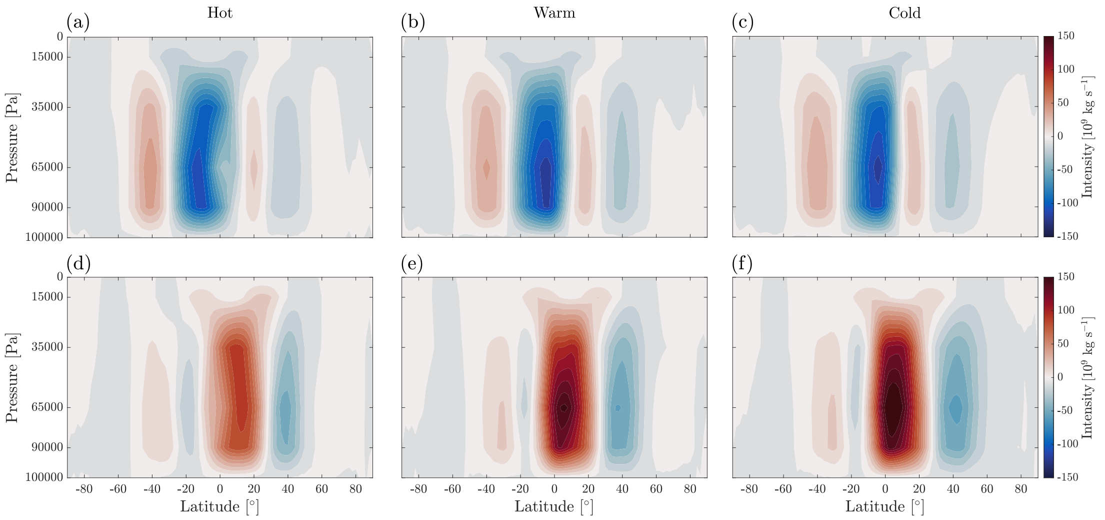

Fig. S1 Seasonal mean atmospheric meridional overturning circulation for the hot state (a,d), the warm state (b,e) and the cold state (c,f). Panels (a-c) and (d-f) corresponds to summer (June-July-August, JJA) and winter (December-January-February, DJF) seasons of the northern hemisphere, respectively.

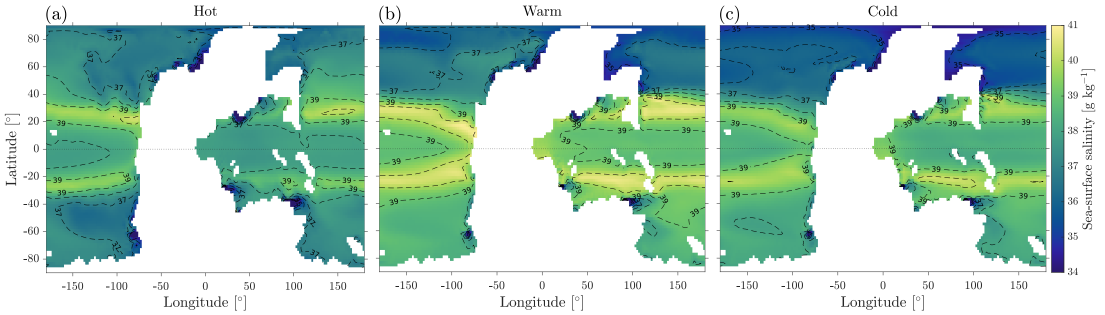

Fig. S2 Sea surface salinity of (a) hot, (b) warm, and (c) cold states. White area corresponds to land.

the main text), giving rise to a symmetrical configuration of the deep-water convection regions at the poles and of the oceanic overturning cells, with strong upwelling at the Equator (Fig. 3d in the main text).

Horizontal and vertical currents are represented in Fig. S3. They are similar in the three attractors, although the subtropical gyres are more symmetrical and intense in the two colder states.

Finally, the cloud cover fraction is shown in Fig. S4. It is larger in the hot state than in the cold state in polar regions and on land. The resulting zonal averages of the net solar and outgoing longwave radiation at TOA are shown in Fig. S5. The main differences occur in the north polar region due to the presence of ice in the cold and warm states.

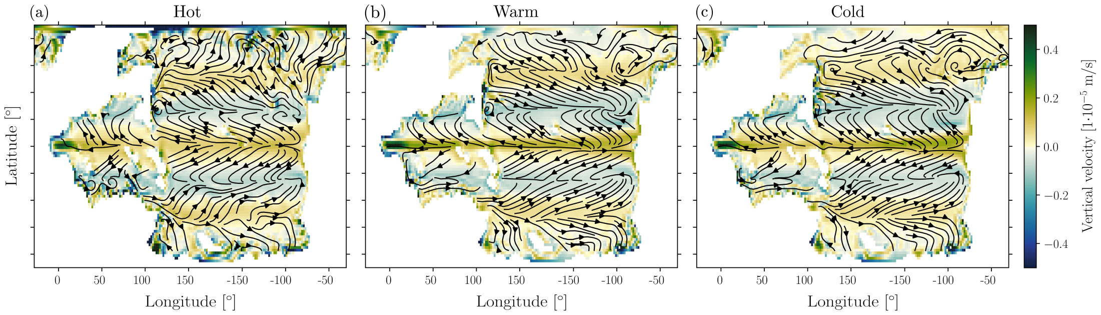

Fig. S3 Surface oceanic circulation in (a) hot, (b) warm and (c) cold states. Black arrows correspond to mean horizontal circulation over the first 50 m. The colorscale represents the vertical velocity: positive (green) corresponds to upwelling, negative (blue) to downwelling.

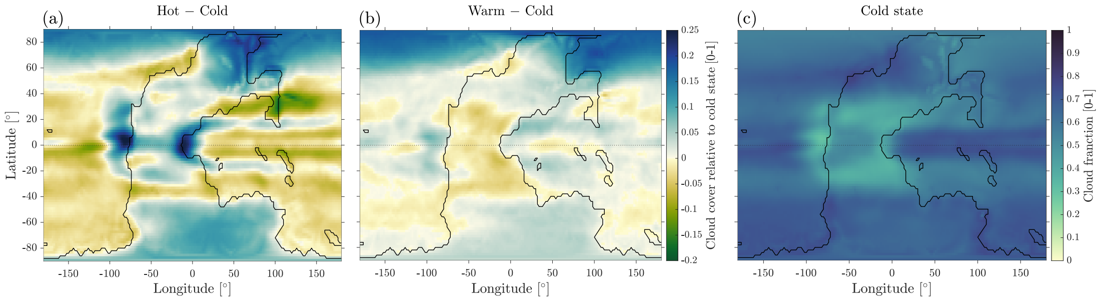

Fig. S4 Cloud cover in difference plots (a) hot *minus* cold; (b) warm *minus* cold; and in the cold state (c).

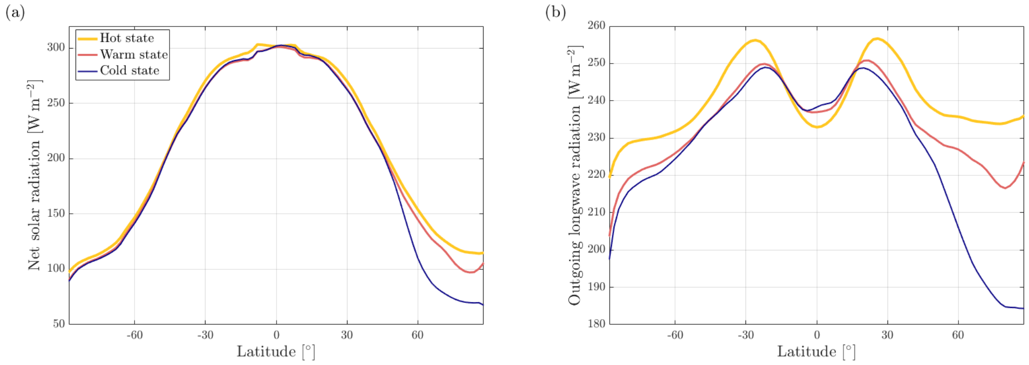

Fig. S5 Zonal averages of (a) net solar radiation and (b) outgoing longwave radiation at TOA for the three attractors.

2 Supplementary material on the construction of the bifurcation diagram

We use Method II described in Brunetti and Ragon (2023) to obtain the bifurcation diagram. In particular, once the three attractors at 320 ppm are found by scanning a large ensemble of initial conditions (see Sections ‘Description

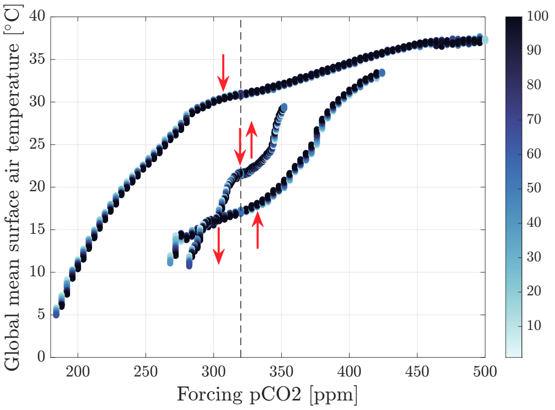

Fig. S6 Bifurcation diagram obtained using Method II in Brunetti and Ragon (2023) by varying the atmospheric pCO_2. The color bar refers to the time (in years) since the last change in the forcing. Red arrows corresponds to tipping point positions along the stable branches.

of three steady states' and 'Methods' in the main text), the atmospheric pCO_2 content is varied by ΔpCO_2 = 2–4 ppm at regular temporal intervals of Δt = 100 yr starting from each attractor, in order to construct the respective stable branches (Fig. S6). Tipping points are found when one of the following three criteria is satisfied (Brunetti and Ragon 2023): (*i*) increased standard deviation of the mean SAT with resepct to its mean value on the attractor; (*ii*) surface energy unbalance; (*iii*) monotonic shift towards another attractor during the time interval Δt.

As can be seen in Fig. S6, at the lower edge of the stable branches of both hot and cold states, the system is attracted towards a colder state. Although we restricted our detailed analysis to the steady states relevant to the Early Triassic, we confirm that at least a colder climate state exists. A waterbelt state is present, for example, at 220 ppm of atmospheric CO_2 content, where the ice extends to $\sim 25°$ latitude and the global mean SAT is around -10 °C (Fig. S7). However, we have not investigated this state further since simulations require long CPU times, and geological data exclude the presence of snowball or waterbelt states in the Early Triassic (Sun et al 2012; Romano et al 2013; Goudemand et al 2019; Widmann et al 2020).

3 Supplementary material on the vegetation cover

Outputs from the MITgcm climate model, which operates on a cubed-sphere grid, must be converted into longitude-latitude coordinates to run the BIOME4 vegetation model. The interpolation between these two grids causes a slight shift in the position of coastlines (due to NaN values over the ocean), which is particularly critical

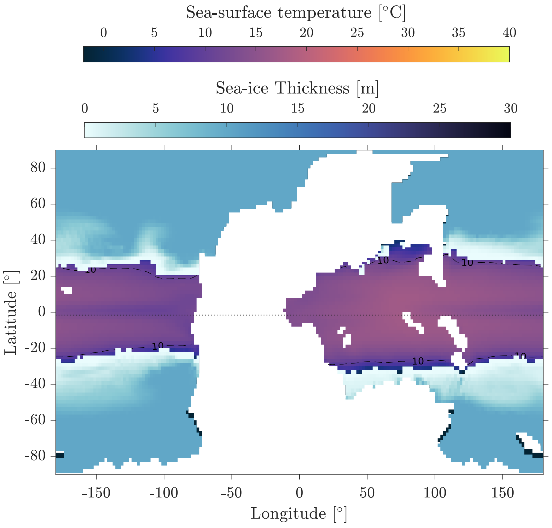

Fig. S7 Sea surface temperature and sea ice extent in the waterbelt state at a global mean surface air temperature of around -10 °C.

for small islands. This is the reason why these small islands are not included in Figs. 6, 7b and 7c in the main text. However, this is only a graphical issue, which does not affect the coupling results.

The coupling between the MITgcm and BIOME4 models is performed as follows:

1. We extract monthly means of precipitation, sunshine (defined as the complement of cloud fraction, an output of MITgcm) and surface air temperature (SAT) from the attractors at pCO_2 = 320 ppm.
2. These averaged values are given as inputs to BIOME4.
3. After running BIOME4, the resulting biomes are converted back into albedo and the fraction of soil covered by vegetation, as reported in Table S1. Note that the present-day ‘grassland’ is replaced by the herbaceous non-graminoid ‘forbland’ because graminoids appeared during the Cenozoic (Gradstein and Kerp 2012). Moreover, biomes with numbers 16 (temperate broadleaved savanna), 18 (boreal parkland), and 28 (land ice) in BIOME4 do not appear in our simulations.
4. We then restart the MITgcm from the same initial state on the attractor with these new boundary conditions, and run it further over 600 yr, which corresponds to an approximate equilibrium.
5. Monthly means are then calculated over the last 300 yr to exclude the first part of the simulation where there is an adjustment to the new vegetation cover, and used as boundary conditions to run BIOME4 again.

Table S1 Albedo and fraction of soil covered by vegetation for each biome obtained as output of BIOME4. The group of biomes used as initial conditions in Fig. 7 in the main text are in italic.

<table>
<tr><th colspan="2">Number and Name</th><th>Albedo [%]</th><th>Vegetation Fraction [%]</th></tr>
<tr><td colspan="2">Cool temperate</td><td rowspan="11">13</td><td rowspan="11">90</td></tr>
<tr><td>1</td><td>Tropical evergreen broadleaf forest</td></tr>
<tr><td>2</td><td>Tropical semi-evergreen broadleaf forest</td></tr>
<tr><td>3</td><td>Tropical deciduous broadleaf forest and woodland</td></tr>
<tr><td>4</td><td>Temperate deciduous broadleaf forest</td></tr>
<tr><td>5</td><td>Temperate evergreen needleleaf forest</td></tr>
<tr><td>6</td><td>Warm-temperate evergreen broadleaf and mixed forest</td></tr>
<tr><td>7</td><td>Cool mixed forest</td></tr>
<tr><td>8</td><td>Cool evergreen needleleaf forest</td></tr>
<tr><td>9</td><td>Cool-temperate evergreen needleleaf and mixed forest</td></tr>
<tr><td>17</td><td>Temperate evergreen needleleaf open woodland</td></tr>
<tr><td colspan="2">Tropical summerwet</td><td rowspan="2">16</td><td rowspan="2">95</td></tr>
<tr><td>12</td><td>Tropical savanna</td></tr>
<tr><td colspan="2">Oceanic influenced dry zone</td><td rowspan="4">19</td><td rowspan="4">50</td></tr>
<tr><td>13</td><td>Tropical xerophytic shrubland</td></tr>
<tr><td>14</td><td>Temperate xerophytic shrubland</td></tr>
<tr><td>15</td><td>Temperate sclerophyll woodland and shrubland</td></tr>
<tr><td>20</td><td>Temperate forbland</td><td>23</td><td>90</td></tr>
<tr><td colspan="2">Tundra</td><td rowspan="8">24</td><td rowspan="8">20</td></tr>
<tr><td>22</td><td>Graminoid and forb tundra</td></tr>
<tr><td>23</td><td>Low and high shrub tundra</td></tr>
<tr><td>24</td><td>Erect dwarf-shrub tundra</td></tr>
<tr><td>25</td><td>Prostrate dwarf-shrub tundra</td></tr>
<tr><td>26</td><td>Cushion-forb tundra</td></tr>
<tr><td>10</td><td>Cold evergreen needleleaf forest</td></tr>
<tr><td>11</td><td>Cold deciduous forest</td></tr>
<tr><td>19</td><td>Tropical forbland</td><td>26</td><td>90</td></tr>
<tr><td colspan="2">Desert</td><td rowspan="3">31</td><td rowspan="3">1</td></tr>
<tr><td>21</td><td>Desert</td></tr>
<tr><td>27</td><td>Barren</td></tr>
</table>

6. This procedure is repeated until convergence between the two models is observed, defined as 1) global SAT does not evolve between two iterations, and 2) the land surface fraction where albedo varies between two iterations is lower than 10%.

To meet the convergence criteria mentioned above, five iterations were needed for the hot state, four for the cold state, and three for the warm state. SAT and percentages of land surface with albedo variations for each iteration are listed in Table S2.

Table S2 Convergence criteria for the offline coupling iterations between BIOME4 and MITgcm. S_{var} corresponds to the surface of land for which albedo is different compared to the previous iteration, rounded to a percent. SAT is averaged over 300 yr, and the error is the inter-annual variability. Bold values highlight satisfied convergence criteria. $B_{surf,o}$ is the global energy budget over the ocean.

	Hot state	Warm state	Cold state
Initial state			
SAT [°C]	30.92 (9)	21.5 (1)	17.1 (1)
$B_{surf,o}$ [W m^{-2}]	0.1 (2)	−0.1 (3)	−0.1 (3)
Iteration 1			
S_{var} [%]	87	87	87
SAT [°C]	32.6 (1)	21.2 (1)	17.1 (1)
$B_{surf,o}$ [W m^{-2}]	0.8 (2)	−0.2 (3)	0.0 (3)
Iteration 2			
S_{var} [%]	37	12	15
SAT [°C]	32.3 (1)	21.76 (9)	18.0 (1)
$B_{surf,o}$ [W m^{-2}]	0.7 (2)	0.0 (3)	0.1 (3)
Iteration 3			
S_{var} [%]	15	**8**	13
SAT [°C]	**32.4 (1)**	**21.8 (1)**	**18.2 (1)**
$B_{surf,o}$ [W m^{-2}]	0.8 (2)	0.0 (3)	0.2 (3)
Iteration 4			
S_{var} [%]	11	-	**6**
SAT [°C]	**32.3 (1)**	-	**18.03 (9)**
$B_{surf,o}$ [W m^{-2}]	0.8 (2)	-	0.1 (3)
Iteration 5			
S_{var} [%]	**8**	-	-
SAT [°C]	**32.4 (1)**	-	-
$B_{surf,o}$ [W m^{-2}]	0.8 (2)	-	-

Seasonal SAT and precipitation are shown in Figs. S8 and S9, respectively, for the hot and cold states when convergence between MITgcm and BIOME4 is attained. The monthly minimum, maximum and mean values for the same quantities are shown in Fig. S10.

Global mean biomass density is estimated for the three attractors at 320 ppm using values for typical ecosystem types reported in Houghton et al (2009). Where several values are provided, the one from Saugier et al (2001) is used. Table S3 shows the correspondence between the biomes used in BIOME4, ecosystem types and biomass densities.

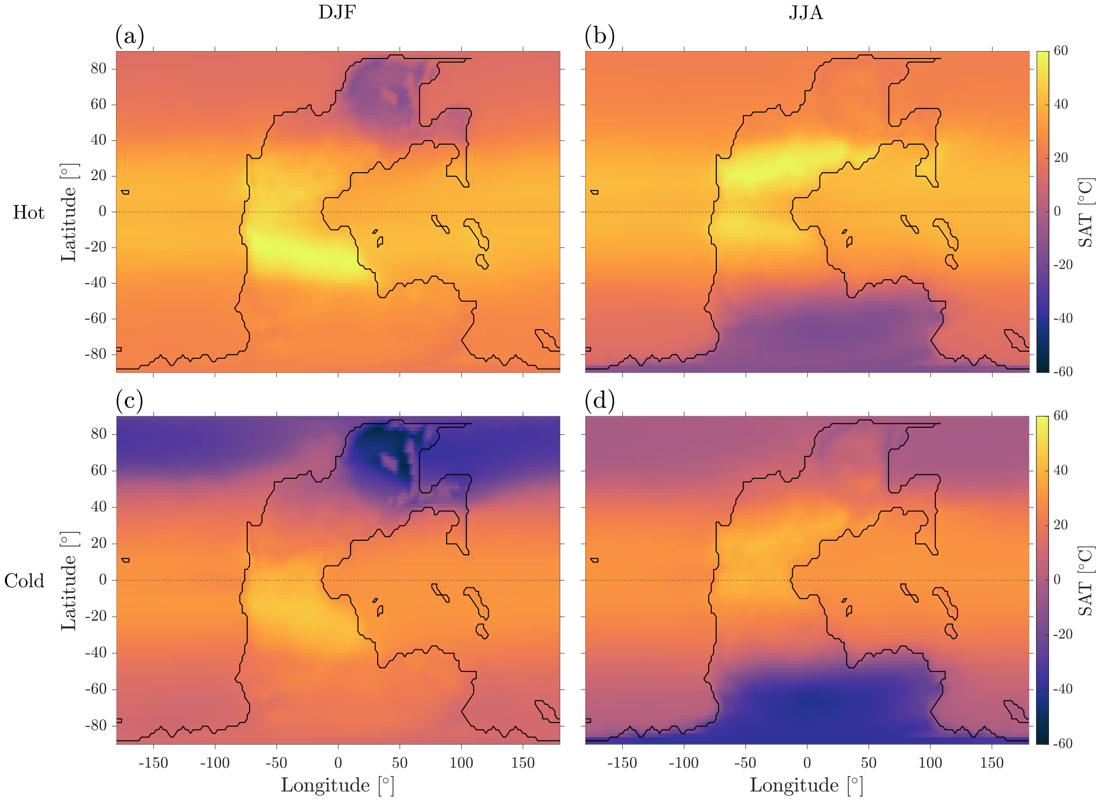

Fig. S8 Seasonal (DJF: a, c; JJA: b, d) surface air temperature in the hot (a-b) and cold (c-d) states when convergence between MITgcm and BIOME4 is attained.

4 Supplementary material on air-sea carbon exchanges

The carbon cycle is partly included by allowing exchanges of carbon between the atmosphere and ocean, as described in Sections ‘Air-sea carbon exchanges’ and ‘Methods’ in the main text. By including this process in the simulations at pCO_2 = 320 ppm and at the edges of the stable branches, we can refine the BD structure (Fig. 5 in the main text). It turns out that this is quite robust, as shown in Fig. S11. However, the range of stability of the warm state almost collapses to a single point around 320 ppm. Thus, this climatic state occupies a very small dynamical phase-space region, which can only be attained from specific initial conditions.

We also estimate the carbon stored in the atmosphere and in the ocean for each attractor (Table S4) and the spatial distribution of the tracers. In particular, we show in Fig. S12 the air-sea flux of CO_2 and the concentration distribution of DIC, DOP and phosphate at the ocean surface, while the oxygen and alkalinity distributions

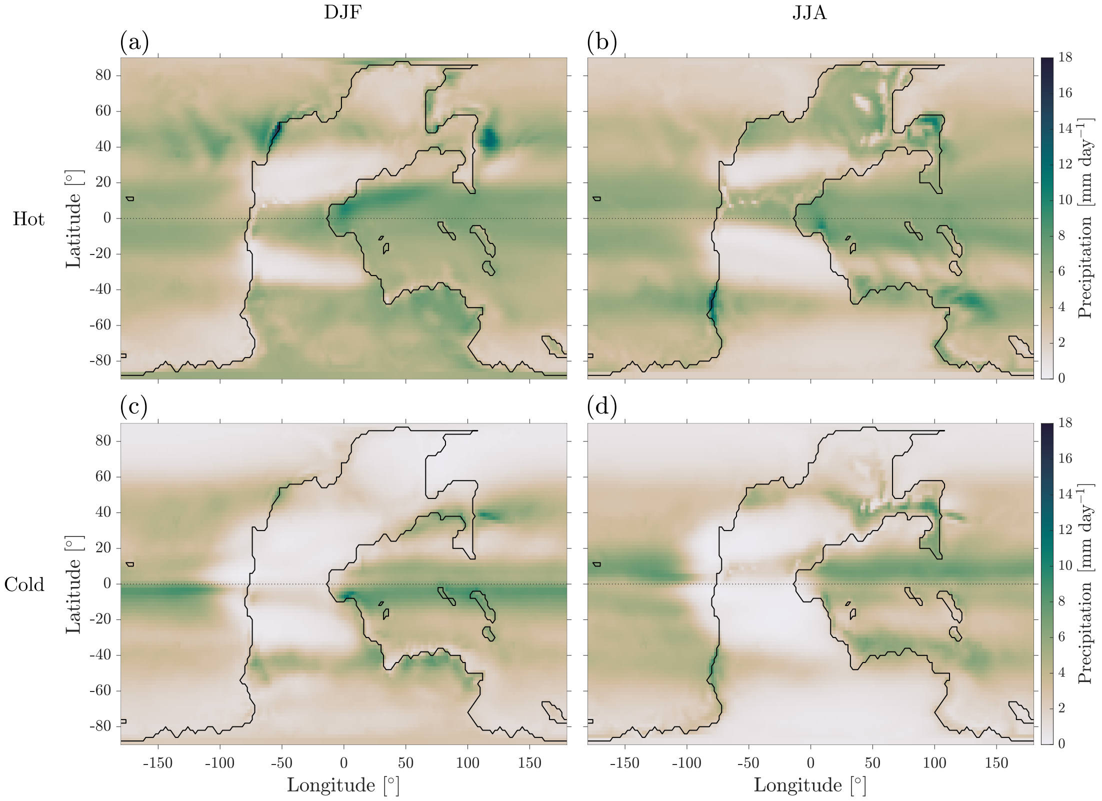

Fig. S9 Seasonal (DJF: a, c; JJA: b, d) precipitation in the hot (a-b) and cold (c-d) states when convergence between MITgcm and BIOME4 is attained.

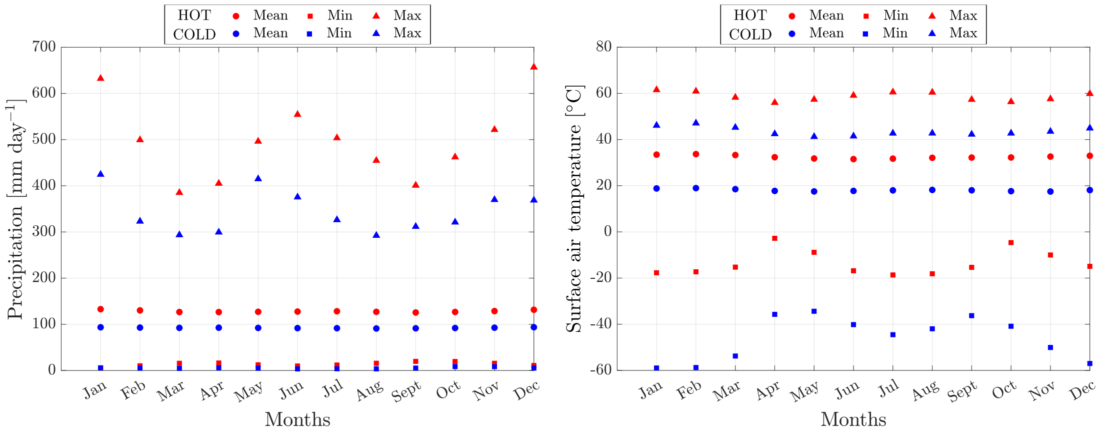

Fig. S10 Monthly mean, minimum and maximum values of precipitation (left) and surface air temperature (right) for the hot and cold states.

Table S3 Correspondence between the biomes used in BIOME4, ecosystem types and biomass density.

Number	Name	Ecosystem type	Biomass density [Mg ha^{-1}]
1	Tropical evergreen broadleaf forest	Tropical forests	390
2	Tropical semi-evergreen broadleaf forest		
3	Tropical deciduous broadleaf forest and woodland		
4	Temperate deciduous broadleaf forest	Temperate forests	270
5	Temperate evergreen needleleaf forest		
6	Warm-temperate evergreen broadleaf and mixed forest		
7	Cool mixed forest	Temperate and boreal forests	160
9	Cool-temperate evergreen needleleaf and mixed forest		
8	Cool evergreen needleleaf forest	Boreal forests	83
10	Cold evergreen needleleaf forest		
11	Cold deciduous forest		
17	Temperate evergreen needleleaf open woodland		
12	Tropical savanna	Tropical savanna and forbland	57
19	Tropical forbland		
13	Tropical xerophytic shrubland	Mediterranean shrublands	120
14	Temperate xerophytic shrubland		
15	Temperate sclerophyll woodland and shrubland		
20	Temperate forbland	Temperate forblands	8
21	Desert	Deserts	7
27	Barren		
22	Graminoid and forb tundra	Arctic tundra	7
23	Low and high shrub tundra		
24	Erect dwarf-shrub tundra		
25	Prostrate dwarf-shrub tundra		
26	Cushion-forb tundra		
28	Land ice	Land ice	0

(not shown) are strongly correlated to sea surface temperature (Fig. 1 in the main text) and sea surface salinity (Fig. S2), respectively, as expected.

Table S4 On the left part, equilibrium SAT when the atmospheric CO_2 content is fixed (at 320 ppm and at the stable-branch edges using the linear fits in Fig. 5). On the right part, new equilibrium values and carbon reservoirs when air-sea carbon exchanges are allowed. Annual mean values are averaged over the last simulated 100 yr.

Fixed CO_2	SAT [°C] with fixed CO_2	SAT [°C] with variable CO_2	Atm. CO_2 [ppm]	Oc. carbon [10^{18} mol]	Atm. + Oc. carbon [10^{18} mol]	Air-sea carbon flux [ppm yr^{-1}]
Hot state						
308 ppm	30.34 (8)	29.7 (1)	307.8 (3)	2.7575 (1)	2.8120 (2)	0.0 (1)
320 ppm	30.90 (7)	30.4 (9)	312.2 (1)	2.7600 (2)	2.8120 (2)	0.00 (5)
440 ppm	36.5 (1)	-	-	-	-	-
Warm state						
320 ppm	21.56 (9)	21.8 (1)	325.1 (4)	2.9018 (2)	2.9594 (1)	-0.01 (4)
328 ppm	22.0 (2)	22.57 (9)	317.2 (3)	2.9051 (1)	2.9612 (1)	0.01 (4)
Cold state						
304 ppm	16.3 (1)	15.7 (1)	310.7 (3)	2.9333 (2)	2.9883 (1)	-0.0 (2)
320 ppm	17.20 (9)	17.26 (9)	320.8 (1)	2.9456 (1)	3.0024 (1)	0.00 (4)
332 ppm	17.9 (1)	17.72 (8)	326.8 (2)	2.94673 (8)	3.0046 (1)	0.01 (4)

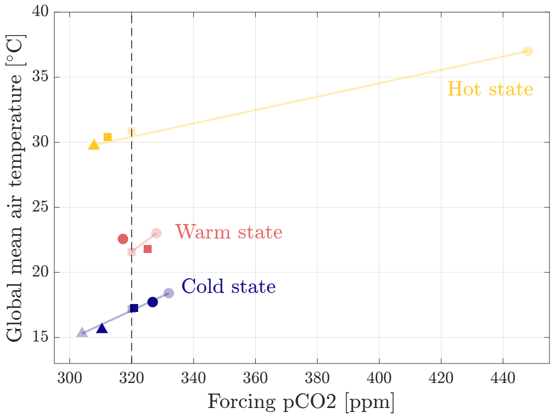

Fig. S11 Bifurcation diagram including air-sea carbon exchanges (dark colors) compared to the version presented in Fig. 5 (shaded colors). Squares correspond to the simulations started at 320 ppm, triangles to the ones at the left edges of the stable branches, and circles to the ones at the right edges.

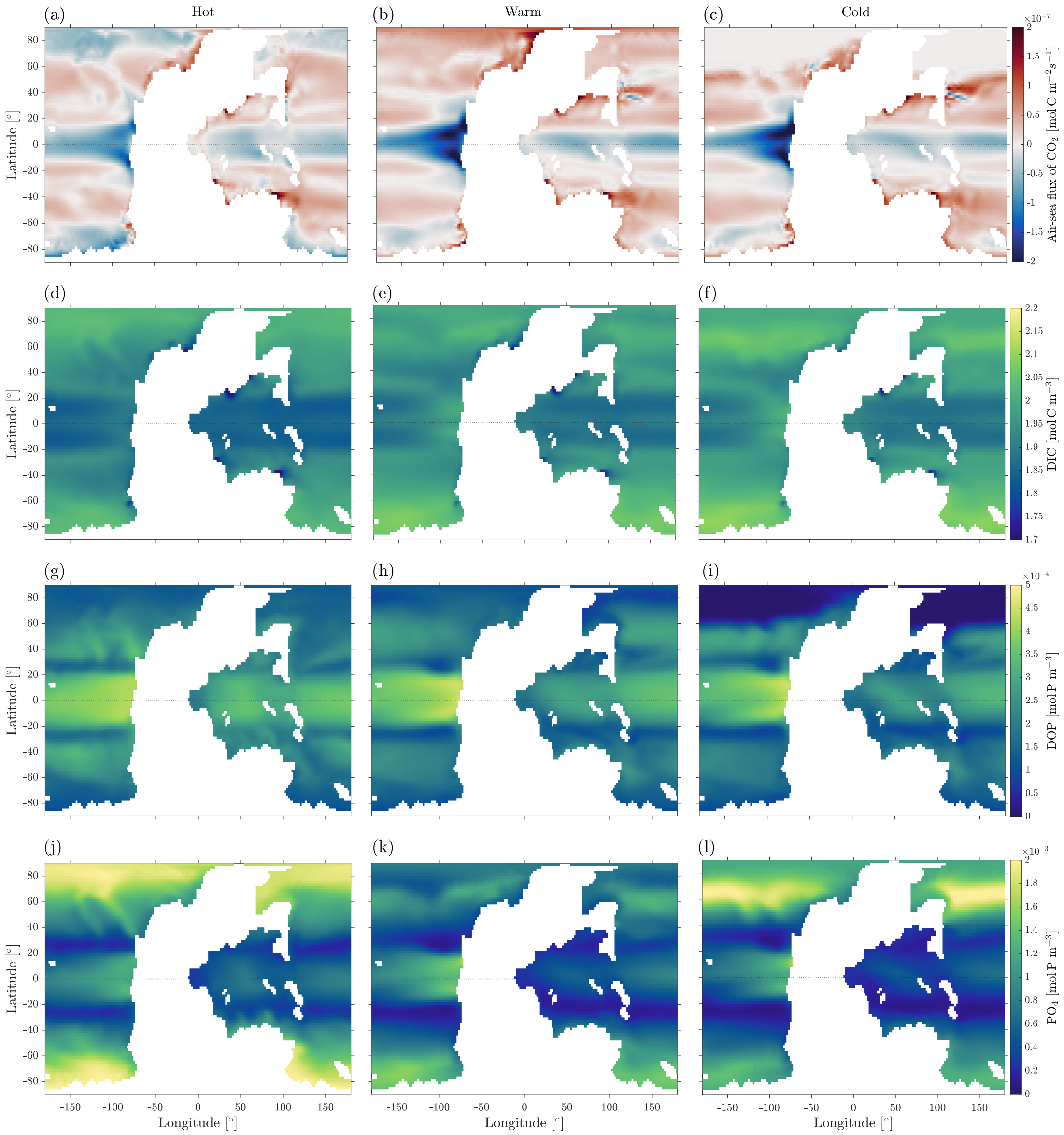

Fig. S12 Distribution of the air-sea flux of CO_2 (a-c), where positive values indicate oceanic uptake from the atmosphere, while negative values indicate outgassing from the ocean. Concentration distributions at the ocean surface of dissolved inorganic carbon (DIC) (d-f), dissolved organic phosphorous (DOP) (g-i) and phosphate (j-l) for the hot (a,d,g,j), warm (b,e,h,k) and cold (c,f,i,l) states. Annual mean values are averaged over the last simulated 30 yr.

References

Brunetti M, Ragon C (2023) Attractors and bifurcation diagrams in complex climate models. Phys Rev E 107:054,214, DOI 10.1103/PhysRevE.107.054214, URL https://link.aps.org/doi/10.1103/PhysRevE.107.054214

Dima IM, Wallace JM (2003) On the seasonality of the hadley cell. Journal of the Atmospheric Sciences 60(12):1522 – 1527, DOI https://doi.org/10.1175/1520-0469(2003)060⟨1522:OTSOTH⟩2.0.CO;2, URL https://journals.ametsoc.org/view/journals/atsc/60/12/1520-0469_2003_060_1522_otsoth_2.0.co_2.xml

Goudemand N, Romano C, Leu M, Bucher H, Trotter JA, Williams IS (2019) Dynamic interplay between climate and marine biodiversity upheavals during the early triassic smithian -spathian biotic crisis. Earth-Science Reviews 195:169–178, DOI https://doi.org/10.1016/j.earscirev.2019.01.013, URL https://www.sciencedirect.com/science/article/pii/S0012825218302563

Gradstein S, Kerp H (2012) Chapter 12 - a brief history of plants on earth. In: Gradstein FM, Ogg JG, Schmitz MD, Ogg GM (eds) The Geologic Time Scale, Elsevier, Boston, pp 233–237, DOI https://doi.org/10.1016/B978-0-444-59425-9.00012-3, URL https://www.sciencedirect.com/science/article/pii/B9780444594259000123

Houghton RA, Hall F, Goetz SJ (2009) Importance of biomass in the global carbon cycle. Journal of Geophysical Research: Biogeosciences 114(G2), DOI https://doi.org/10.1029/2009JG000935, URL https://agupubs.onlinelibrary.wiley.com/doi/abs/10.1029/2009JG000935, https://agupubs.onlinelibrary.wiley.com/doi/pdf/10.1029/2009JG000935

Lindzen RS, Hou AV (1988) Hadley circulations for zonally averaged heating centered off the equator. Journal of Atmospheric Sciences 45(17):2416 – 2427, DOI https://doi.org/10.1175/1520-0469(1988)045⟨2416:HCFZAH⟩2.0.CO;2, URL https://journals.ametsoc.org/view/journals/atsc/45/17/1520-0469_1988_045_2416_hcfzah_2_0_co_2.xml

Romano C, Goudemand N, Vennemann TW, Ware D, Schneebeli-Hermann E, Hochuli PA, Brühwiler T, Brinkmann W, Bucher H (2013) Climatic and biotic upheavals following the end-permian mass extinction. Nature Geoscience 6(1):57–60

Saugier B, Roy J, Mooney HA (2001) Estimations of global terrestrial productivity. Terrestrial global productivity pp 543–557

Sun Y, Joachimski MM, Wignall PB, Yan C, Chen Y, Jiang H, Wang L, Lai X (2012) Lethally hot temperatures during the early triassic greenhouse. Science 338(6105):366–370, DOI 10.1126/science.1224126, URL https://www.science.org/doi/abs/10.1126/science.1224126, https://www.science.org/doi/pdf/10.1126/science.1224126

Widmann P, Bucher H, Leu M, Vennemann T, Bagherpour B, Schneebeli-Hermann E, Goudemand N, Schaltegger U (2020) Dynamics of the largest carbon isotope excursion during the early triassic biotic recovery. Frontiers in Earth Science 8, DOI 10.3389/feart.2020.00196, URL https://www.frontiersin.org/articles/10.3389/feart.2020.00196
